# Restless Legs Syndrome: From Pathophysiology to Clinical Diagnosis and Management

**DOI:** 10.3389/fnagi.2017.00171

**Published:** 2017-06-02

**Authors:** Shiyi Guo, Jinsha Huang, Haiyang Jiang, Chao Han, Jie Li, Xiaoyun Xu, Guoxin Zhang, Zhicheng Lin, Nian Xiong, Tao Wang

**Affiliations:** ^1^Department of Neurology, Union Hospital, Tongji Medical College, Huazhong University of Science and TechnologyWuhan, China; ^2^Department of Psychiatry, Harvard Medical School, BelmontMA, United States; ^3^Division of Alcohol and Drug Abuse, Mailman Neuroscience Research Center, McLean Hospital, BelmontMA, United States

**Keywords:** clinical presentation, diagnostic criteria, iron deficiency, dopamine, A11 cell group, treatment, augmentation, loss of efficacy

## Abstract

Restless legs syndrome (RLS), a common neurological sensorimotor disorder in western countries, has gained more and more attention in Asian countries. The prevalence of RLS is higher in older people and females. RLS is most commonly related to iron deficiency, pregnancy and uremia. The RLS symptoms show a significant circadian rhythm and a close relationship to periodic limb movements (PLMs) in clinical observations, while the pathophysiological pathways are still unknown. The diagnostic criteria have been revised in 2012 to improve the validity of RLS diagnosis. Recent studies have suggested an important role of iron decrease of brain in RLS pathophysiology. Dopaminergic (DA) system dysfunction in A11 cell groups has been recognized long ago from clinical treatment and autopsy. Nowadays, it is believed that iron dysfunction can affect DA system from different pathways and opioids have a protective effect on DA system. Several susceptible single nucleotide polymorphisms such as BTBD9 and MEIS1, which are thought to be involved in embryonic neuronal development, have been reported to be associated with RLS. Several pharmacological and non-pharmacological treatment are discussed in this review. First-line treatments of RLS include DA agents and α2δ agonists. Augmentation is very common in long-term treatment of RLS which makes prevention and management of augmentation very important for RLS patients. A combination of different types of medication is effective in preventing and treating augmentation. The knowledge on RLS is still limited, the pathophysiology and better management of RLS remain to be discovered.

## Background

The symptoms of the RLS were first described by Willis (1685) and then published by Ekbom (1960). Despite being introduced hundreds of years ago, it’s still a poorly recognized disorder because of the unclear pathophysiology and relatively low morbidity, resulting in limited recognition by primary care physicians and common misdiagnosis and under-diagnosis. RLS is considered as a common neurological sensorimotor disorder that manifests as an irresistible urge to move the body to relieve the uncomfortable sensations. There’s a significant circadian rhythm of the RLS, as it commonly worsens at night.

## Epidemiology

It has been concluded that the prevalence rate ranged from 3.9 to 15% of the general population (Ohayon et al., 2012) from recent epidemiologic analyses of different countries. The estimated prevalence of the RLS is around 7–10% in Caucasians (Ohayon et al., 2012) while there is a much lower incidence ranging from 0.1 to 12% among Asian population (Cho et al., 2009; Tsuboi et al., 2009; Chen et al., 2010; Panda et al., 2012; Shi et al., 2015). Compared with other countries, RLS is much more common in western countries. As numerous studies in western countries indicated the prevalence of 10, 10–15, and 5.5% in United States, Canada, and Europe (United Kingdom, Spain, Germany, Italy), respectively (Phillips et al., 2000; Ohayon and Roth, 2002). On the other hand, studies from Asian countries showed a pretty low prevalence rate of RLS. The first Indian population study on RLS revealed a prevalence of 2.9% (Panda et al., 2012) while a prevalence of 0.96% among inhabitants of Ajimu in Japan who were older than 65 years old (Tsuboi et al., 2009). Another RLS study in Shanghai, China revealed a prevalence rate of 1.4% among 2941 eligible individuals older than 18 years old (Shi et al., 2015). The significant difference between different ethnic populations may be due to different genetic background, ethnicity, geography, and environmental influences including natural environment and diet habits. The different populations targeted, research methodologies and diagnosis criteria used may contribute to this result as well.

Most surveys have concluded that the prevalence and severity of the RLS both increase with age (Phillips et al., 2000; Ohayon and Roth, 2002), suggesting that the neurodegenerative process may play an important role in RLS. Life style of older people and the senile changes including cardiovascular changes and metabolism changes is also related to RLS (Koh et al., 2015; Cassel et al., 2016). In adults, the incidence of RLS is twice as high in women than in men (Berger et al., 2004), which may result from the hormones such as estrogen and progesterone and different social roles.

The age of onset varies widely from childhood to over 90 years of age. Though most patients in clinical practice are middle-aged or older, juvenile onset is not rare. 38–45% of adult patients complain about first symptoms before the age of 20 (Walters et al., 1996; Montplaisir et al., 1997). A study in 2007 found rates of 1.9 and 2.0% in 8–11 years old and 12–17 years old children and adolescence, respectively. Another study using a different diagnostic criteria for RLS in children that published in 2003 revealed a prevalence of 5.9% (Kotagal and Silber, 2004). A newest pediatric RLS diagnostic criteria that include consideration of typical words used by children, differential diagnosis and comorbidity was published in 2013 (Picchietti et al., 2013). But no gender difference is found in pediatric RLS (Per et al., 2017). This gender difference in adult but not in children might be due to pregnancy because nulliparous women have about the same prevalence of RLS as males (Berger et al., 2004). The different classifications of RLS including primary and secondary RLS may also contribute to these findings. Sleep, mood, cognition, and quality of life are significantly affected in pediatric RLS patients (Picchietti and Picchietti, 2010). And ADHD, depressive symptoms, and anxiety are very common comorbidities of pediatric RLS patients (Pullen et al., 2011).

## Clinical Presentations and Diagnostic Criteria

### Clinical Presentations

Restless legs syndrome manifests as an overwhelming urge to move the body to relieve the uncomfortable sensations, primarily when resting, sitting, or sleeping. The uncomfortable feelings are always described by the patients as “creeping, crawling tingling, tingling, pulling, or painful” deep inside the limbs (Trenkwalder et al., 2005), unilaterally or bilaterally occurring with the knees, the ankles or even the whole lower limbs (Trenkwalder et al., 2005). Sometimes even the phantom limbs can be involved (Skidmore et al., 2009). Commonly it affects the patient’s sleep. Insomnia is the most common reason for a patient with RLS to search for consult in clinical practice. The most common bedtime problems caused by RLS is difficulty initiating sleep (Mohri et al., 2008). It has a significant circadian pattern which presents as worsening symptoms in the evening and short remission in the morning after waking up (Kushida et al., 2007). The longer the course of the disease, the more likely the symptoms affect the arms or the other places of the body besides the legs. It’s not uncommon that patients developed arm restless syndrome with progressive disease (Freedom and Merchut, 2003). Movement such as walking, stretching, or bending the legs relieves the discomfort at least temporarily and partially (Trenkwalder et al., 2005).

Restless legs syndrome affects the patient’s HRQoL to different degrees according to various severities of the symptoms. HRQoL is measured by 36-Item Short Form Health Survey (SF-36), a 36 item survey used to construct eight scales among physical and mental health and health transition. Most patients have mild symptoms, only 11.9% of them seek for consult, while about 3.4% of all the patients need drug treatment (Hening, 2004). The main morbidities except for extreme discomfort were sleep loss and disruption of normal activities (Trenkwalder et al., 2005). Patients with mild or moderate symptoms manifested discomfort with less frequency, lower severity, and less influence of the symptoms on their sleep. Comparing with the general population, patients with severe or very severe RLS symptoms always reported apparent deficits (10–40 points on 100-points scales) in physical functioning, bodily pain, general health, vitality, social functioning, role-physical, and role-emotion (Trenkwalder et al., 2005). With deficient sleep at night, severely affected patients might complain about having difficulty in their daily life including their jobs and social activities (Kushida et al., 2007). A significant higher probability of 25% with ADHD were found in RLS patients compared to general population (Pullen et al., 2011). On the other hand, 20% of ADHD patients meet criteria for RLS as well (Zak et al., 2009; Yilmaz et al., 2011). Pathophysiological pathways of ADHD remains poorly understood, but the most popular theory is the dopamine deficit theory, which could be a shared pathway with RLS (Swanson et al., 2007). *BTBD9*, a RLS risk allele, may be related to certain subtypes of ADHD, which responds well to iron supplementation treatment (Schimmelmann et al., 2009). Disruption of sleep length and quality, daytime alertness can contribute to depression and anxiety. Increasing sympathetic tone due to PLMS may lead to cardiovascular disease and high blood pressure (Stevens, 2015).

Different from in adults, pain is a common presentation in pediatric RLS. 45% of children use the terms pain and hurts or hurting which makes growing pains a common misdiagnosis in pediatric RLS (Picchietti et al., 2013). And children always use their own words like “need to move, want to move and got to kick (Picchietti et al., 2011)” to describe “urge”. ADHD is also a very common comorbidity in pediatric RLS. Except for bad impact on sleep, mood and cognition, behavioral and educational changes are very common in pediatric RLS (Picchietti et al., 2013). It might be due to disruption of homework and ability to concentrate.

### Associated Features

#### Circadian Rhythm

Both sensory and motor symptoms show significant circadian rhythm in RLS, which display as a peak at similar time at night. A study showed the increase in melatonin secretion to be the only changes preceding the sensory and motor symptoms in RLS patients, indicating melatonin might affect the symptoms by its inhibitory effect on dopamine secretion in central nervous system (Michaud et al., 2004). The intensity of RLS symptoms peaks on the falling phase of the core body temperature, another endogenous marker of circadian rhythm, while it decreases when core temperature increases (Hening et al., 1999; Barriere et al., 2005).

Previous studies have shown that plasma dopamine and its metabolites changes with circadian rhythm not only just in humans (Sowers and Vlachakis, 1984), but also in CSF in primate animals (Perlow et al., 1977) and in the striatum of rat (Schade et al., 1995; Castaneda et al., 2004). Another study indicated that the dopamine receptor responsiveness is modulated by the circadian rhythm at the level of spinal cord in decapitated *Drosophila melanogaster* (Andretic and Hirsh, 2000). Moreover, it has been revealed that sensitivity of dopamine receptors increased at night at the level of tubero-infundibular-dopaminergic system (Garcia-Borreguero et al., 2004). Additionally, a circadian variation of serum iron paralleled the CSF dopamine, as well as the severity of symptoms (Garcia-Borreguero et al., 2004). However, it is unclear whether the brain iron concentrations changes would follow this pattern.

#### Periodic Limb Movement Disorder

Periodic limb movement disorder, previously known as nocturnal myoclonus, is defined as involuntary movements of the patient’s limb or torso during awake or sleep which the patient is not aware of, different from the voluntary movement of the limb to relieve the discomfort in RLS patients (Hening et al., 1999; Trenkwalder et al., 2005). Nevertheless, it’s a very common phenomenon in RLS patients. A previous study indicated that PLMS was found in around 80% of RLS patients (Montplaisir et al., 1997). On the contrary, not a large percentage of patients having PLMS presented RLS. PLMS, which is not a specific feature for RLS patients, can be associated to many other conditions. The PSG is usually employed to measure the movements, while actigraphy is a helpful method for diagnosing and measuring PLMW or PLMS. PLMS is diagnosed on PSG by at least continuous four muscle contractions lasting 0.5–10 s and recurring during intervals of 5–90 s. The minimum amplitude of a leg movement event is an 8 μV increases in EMG voltage above resting EMG (Iber et al., 2007) in diagnostic criteria for PLMD.

A movement in PLMS starting in sleep can continue when waking up and vice versa. On the other hand, arousals happening before or during a movement event do not change the assessment of that event (Iber et al., 2007). Immobilization test is to measure PLMW in which the patient is asked to lie perfectly still. The PSG records the time the patient can stay still and the limb movements during an hour. It can be used to quantify the severity of RLS, to follow up the patient’s course of disease and to monitor treatment response (Trenkwalder et al., 2005).

### Diagnostic Criteria

The diagnostic criteria have experienced a lot of improvements and revisions in the history, including the earliest informal Ekbom’s “criteria” for RLS in 1960, then DCSAD restless legs DIMS or DOES syndrome – essential features in 1979, ICSD diagnostic criteria for RLS in 1990, IRLSSG “minimal” criteria for diagnosis of RLS in 1995 and NIH/IRLSSG (NIH) “essential” criteria for diagnosis of RLS in 2003 (Allen et al., 2014). On the basis of previous diagnostic criteria, the four essential diagnostic criteria of RLS published by NIH/IRLSSG in 2003 emphasized the importance of the urge to move the legs in diagnosing RLS. The four essential criteria are shown below (**Table 1**).

**Table 1:** 2003 NIH/IRLSSG diagnostic criteria.

(1) The urge to move the legs are usually accompanied by or caused by uncomfortable sensations deep in the legs. (2) The above symptoms begin or worsen when resting or inactivity such as lying or sitting. (3) The symptoms can be partially or totally relieved by movement, such as walking or stretching. (4) The symptoms are worse in the evening or at night rather than during the day, or they only occur in the evening or at night.

Although, 2003 NIH/IRLSSG diagnostic criteria have defined RLS in a much more detailed way than those previous criteria. The same disadvantage still exist in this criteria, RLS “mimics” can’t be excluded according to this diagnostic criteria. Such as conditions like cramps, positional discomfort and local leg pathology. Then the diagnostic criteria were recently revised again by the IRLSSG in 2012 (International Restless Legs Syndrome Study Group, 2012). Comparing with 2003 NIH/IRLSSG diagnostic criteria, 2012 revised RLS diagnostic criteria have added an important essential criterion, noting that RLS should be differentiated with other conditions with similar symptoms such as myalgia, venous stasis, leg edema, arthritis, habitual foot tapping, and so on (International Restless Legs Syndrome Study Group, 2012). 2012 revised RLS diagnostic criteria also stated the stipulation of clinical course and clinical significance of RLS as presented (International Restless Legs Syndrome Study Group, 2012). The newest diagnostic criteria (**Table 2**) is much more rigorous than 2003 IRLSSG diagnostic criteria.

**Table 2:** 2012 revised IRLSSG diagnostic criteria (International Restless Legs Syndrome Study Group, 2012).

Essential diagnostic criteria	
(1) An urge to move the legs usually but not always accompanied by, or felt to be caused by, uncomfortable and unpleasant sensations in the legs. (2) The urge to move the legs and any accompanying unpleasant sensations begin or worsen during periods of rest or inactivity such as lying down or sitting. (3) The urge to move the legs and any accompanying unpleasant sensations are partially or totally relieved by movement, such as walking or stretching, at least as long as the activity continues. (4) The urge to move the legs and any accompanying unpleasant sensations during rest or inactivity only occur or are worse in the evening or night than during the day. (5) The occurrence of the above features is not solely accounted for as symptoms primary to another medical or a behavioral condition (e.g., myalgia, venous stasis, leg edema, arthritis, leg cramps, positional discomfort, habitual foot tapping).	
**Specifiers for clinical course of RLS/WED**	
(A) Chronic-persistent RLS	Symptoms occurring at least twice a week on average for the past year when not treated.
(B) Intermittent RLS	Symptoms occurring less than twice a week on average for the past year when not treated.
**Specifiers for clinical significance of RLS/WED**	
The symptoms of RLS/WED cause significant distress or impairment in social, occupational, educational or other important areas of functioning by their impact onsleep, energy/vitality, daily activities, behavior, cognition, or mood.	

Specifier for clinical significance of RLS emphasizes that the influence of RLS on the patient’s function in social, occupational, educational, or other important areas should be evaluated. Different from in adults, functional consequences of RLS in children are mainly behavioral and educational domains (Arbuckle et al., 2010). The new diagnostic criteria set up a more rigorous method to ascertain a RLS case with more specific criteria and excluding standards. It has improved the validity of RLS diagnosis. On the other hand, it helps to classify the patient’s clinical course and clinical significance which helps the physician to better monitor the progression of RLS and support better cases for research samples (Allen et al., 2014). The specifier for clinical course does not apply for pediatric RLS or special cases of RLS secondary to pregnancy or medication (Picchietti et al., 2013). Generally, mild RLS severity with no family history, and young age at RLS onset are predictors of RLS remission. While most patients with severe RLS show a chronic clinical course (Lee et al., 2016).

Periodic limb movement during sleep is a very identified sign in RLS patients. It is found that 80–89% of RLS patients have excessive PLMS than same aged people (Montplaisir et al., 1997). PLMS is not very specific for RLS since it is a frequent condition among adults aged over 45 years (Hornyak et al., 2007). When PLMS present in a pattern different than expected for age excluding the states of other disease or medication, PLMS can support the diagnosis of RLS.

Considering that children might not understand the term “urge”, simple straightforward prompts should be asked, like “Do your legs bother you?” or “Do your legs bother you at night?” And common descriptions used by children about RLS sensations are “need to move, want to move and got to kick (Picchietti et al., 2011)”. RLS mimics like ADHD, sore leg muscles, growing pains and dermatitis should be carefully considered when diagnosing pediatric RLS (Picchietti et al., 2013).

Comparing with the previous diagnostic criteria, 2012 revised RLS diagnostic criteria is the only criteria that developed by a large international number of RLS clinical and research experts through an interdisciplinary, international and evidence-based approach which ensure it to be a world-wide consensus criteria which reduces the risk of cultural bias and avoids arbitrary and improve the validity. The specifiers for clinical course and significance are able to provide a method to define different target population which would help clinicians and researchers to offer better prevention and treatment strategies for specific groups of patients and to better elucidate etiopathogenesis (Allen et al., 2014).

On the other hand, some disadvantages still exist since the diagnostic criteria for RLS are subjective, some more objective and reliable diagnostic criteria need to be brought up for a further diagnosis and classification of RLS, such as biological markers, genetic features, PLMS measuring, polysomnography, and actigraphy changes. Some new tools are to be developed for standardize case ascertainment (Allen et al., 2014). A case-control study in Germany reveals that inositol metabolites increased specifically in RLS patients (Schulte et al., 2016). This might be a discovery approach using serum metabolite profiling in RLS.

### Classification

The RLS includes two groups in general: primary RLS and secondary RLS.

Primary RLS is considered to be idiopathic when the cause is truly unknown. Among the idiopathic RLS, 40.9–92% of whom had a family history of RLS, indicating the important role of genetic factors in developing RLS (Winkelman et al., 1996; Winkelmann et al., 2002; Tison et al., 2005).

Most secondary RLS cases have an onset after 40 years old. Secondary RLS are those associated with a variety of neurological disorders, iron deficiency, pregnancy, or chronic renal failure (Winkelman et al., 1996; Curgunlu et al., 2012; Srivanitchapoom et al., 2014). A study in Turkey revealed that severity of RLS symptoms is very closely related to low ferritin level (Curgunlu et al., 2012). In the earliest studies of RLS, some revealed that 25% of RLS patients have iron deficiency condition. The other correlated factors were diabetic peripheral neuropathy (Zobeiri and Shokoohi, 2014), painful neuropathies (Rutkove et al., 1996), ADHD (Roy et al., 2015), migraine (Zanigni et al., 2014), AS (Tekatas and Pamuk, 2015), leprosy (Padhi and Pradhan, 2014), inflammatory chronic demyelinating neuropathies like multiple sclerosis (Deriu et al., 2009) and Guillain–Barré syndrome (Marin et al., 2010), thyroid disease (Rodriguez Martin et al., 2015), poliomyelitis (Kumru et al., 2014), chronic venous disorder (McDonagh et al., 2007), autoimmune disease including Sjögren’s syndrome (Theander et al., 2010), rheumatoid arthritis (Hening and Caivano, 2008), inflammatory bowel disease (Becker et al., 2015), and Crohn’s disease (Hoek et al., 2015). Epidemiology studies also showed that the prevalence of RLS significantly increased in post-stroke patients, mainly in patients whose stroke topography lied on pyramidal tract and the basal ganglia-brainstem axis which were primarily involved in motor functions (Sechi and Sechi, 2013). Some certain drugs can cause or worsen RLS symptoms, such as psycotropics like antidepressants and neuroleptics, dopaminergic drugs, and some other drugs (Giudice, 2010) such as cumulative dopaminergic agonists effects in Parkinson’s disease patients (McDonagh et al., 2007). A study revealed that the strongest evidence for drug-induced RLS are for the following: escitalopram, fluoxetine, L-dopa/carbidopa and pergolide, L-thyroxine, mianserin, mirtazapine, olanzapine, and tramadol (Hoque and Chesson, 2010).

According to the onset age of the symptoms, RLS is divided into early-onset RLS and late-onset RLS. Early-onset RLS refers to those who firstly have the symptoms before 45 years old. While late-onset RLS patients have the symptoms from or after 45 years old. A higher familial history rate was found in early-onset RLS comparing to late-onset RLS (Kotagal and Silber, 2004). Various clinical courses with periodic remissions are common in early-onset RLS. While a chronic progressive clinical course with more severe symptoms are seen in late-onset RLS. Pediatric RLS are always misdiagnosed as “growing pain”. Current researches demonstrate a relatively iron deficiency and renal failure to be exacerbating factors for pediatric RLS (Kotagal and Silber, 2004; Davis et al., 2005; Applebee et al., 2009; Sinha et al., 2009). Commonly pediatric RLS should be differentiated from ADHD while only RLS patients presents the need to move because of leg discomfort but not difficulty “sitting still” (Trenkwalder et al., 2005).

## Differential Diagnosis

Very common conditions which should be differentiated with RLS include leg cramps, positional discomfort, local leg injury, arthritis, leg edema, venous stasis, peripheral neuropathy, radiculopathy, habitual foot tapping/leg rocking, anxiety, myalgia, and drug-induced akathisia (Allen et al., 2014). They can mimic RLS in different ways. Leg cramps are presented as knot of the muscle. Positional discomfort can be relieved by a positional shift. Arthritis patients have a limitation of the joints or joint erythema. Myalgias present as muscle soreness. Numbness happen to neuropathy patients as well as RLS patients, and both venous stasis and leg edema can manifest as swelling in the limbs (Davis et al., 2005; Applebee et al., 2009; Sinha et al., 2009; Karroum et al., 2012). Less common differential diagnostic conditions included myelopathy, myopathy, vascular or neurogenic claudication, hypotensive akathisia, orthostatic tremor, painful legs, and moving toes (Allen et al., 2014). Differential diagnosis is really important to RLS patients for their further treatment. Therefore clinical physicians have created additional diagnostic questionnaires and scales to improve the diagnosis of RLS in clinical practice (Popat et al., 2010). RLS-NIH questionnaire, developed in 2002 with three mandatory questions, showed sensitivity and specificity of 86 and 45% respectively (Popat et al., 2010). On the basis of this, an the RLS-EXP showed the sensitivity and specificity of 81 and 73% respectively (Popat et al., 2010). The CH-RLSq is more commonly used in clinical practice and by researchers.

## Secondary RLS

### Iron Deficiency

Early in 1953, Nordlander (1953) first proposed that iron deficiency might be an important part of the pathophysiological process in RLS which was supported by consistent prevalence studies and recent pharmacological researches. Low serum iron levels (normal range: 50–170 μg/dL for men; 65–176 μg/dL for women; 50–120 μg/dL for children) presented in 25% of patients with severe RLS (Ekbom, 1960). While 43% of patients complaining of “leg restless” were found to be in the condition of iron deficiency (Matthews, 1976). The severity of the symptoms was found to be correlated with serum ferritin levels (normal range: 15–200 ng/mL for men; 12–150 ng/mL for women; 7–140 ng/mL for children) (O’Keeffe et al., 1994; Mizuno et al., 2005). Numbers of pharmacological studies of iron supplement for RLS gained therapeutic effects (Nordlander, 1953). CSF biological studies showed lower iron and ferritin levels in RLS patients, along with higher transferrin levels (Mizuno et al., 2005). However, a current study has suggested that in normal circumstances, the brain does not respond to peripheral variations in iron status (Ward et al., 2014). This might explain why some RLS patients had a normal or over-loaded serum iron level while their CSF iron level was decreased. Various medical imaging studies including ultrasound studies and MRI studies revealed a decreased iron levels in the substantia nigra and putamen, especially in the very severe patients (Schmidauer et al., 2005; Moon et al., 2014). Other studies demonstrated iron decrease in the red nucleus, thalamus and the pallidum (Haba-Rubio et al., 2005; Rizzo et al., 2013). However, MRI was not capable of locating the iron deficiency condition to particular cells up to now. Autopsy studies also reported a decrease in iron concentration in the substantia nigra (Moon et al., 2014).

### Pregnancy

15–25% of pregnant women have RLS in Western countries according to prevalence studies (Lee et al., 2001; Neau et al., 2010). A peak prevalence of RLS in pregnant women mainly occurred in the third trimester and gained a remission by 1 month after delivery (Ismailogullari et al., 2010). Surveys manifested that nulliparous women were at the same risk of RLS as same aged men, while the risk for women were increased after one pregnancy (OR 1.98) and two pregnancies (OR 3.04), even more after three or more pregnancies (OR 3.57) (Berger et al., 2004; Pantaleo et al., 2010). These findings may explain the gender difference in RLS prevalence. The reason why pregnant women have higher risk of having RLS remains unknown. One reason is that an increased demand of iron in pregnant women results in relative iron deficiency. The other established factors included hormonal status (prolactin, progesterone and estrogen), folate deficiency and stretch or compression of nerves due to fetal growth conflicting (Pantaleo et al., 2010; Pereira et al., 2013). Psychomotor behavioral change during the last weeks of pregnancy might also contribute to the symptoms. Anxiety, insomnia, and fatigue are always associated with pregnancy in the last trimester.

### End-Stage Renal Disease Patients on Hemodialysis

Studies have revealed approximate prevalence of 20–30% RLS in hemodialysis than the prevalence of 3.9–15% among the general populations (Ohayon et al., 2012). A study including 166 patients in Serbian showed 22.7% of patients on hemodialysis were under the condition of RLS (Nikic et al., 2007). Other studies indicated a prevalence of 14.8% among 176 patients on hemodialysis in Brazil (Goffredo Filho et al., 2003) and a prevalence of 37.4% in 163 patients on hemodialysis in Iran (Rohani et al., 2015). Some of the studies revealed a predominance of female patients with RLS rather than male patients on hemodialysis (Al-Jahdali et al., 2009; La Manna et al., 2011; Haider et al., 2014), while a recent study in Saudi displayed no statistically difference between two genders (Wali and Alkhouli, 2015). Most of the RLS patients among end-stage renal disease indicated moderate to severe symptoms compared to most mild RLS symptoms among general populations (Wali and Alkhouli, 2015). Iron deficiency in end-stage renal disease patients may lead to anemia and affect the dopamine metabolism, contributing to RLS (Nikic et al., 2007), and uremia-related peripheral neuropathy and high serum calcium can be part of the physiology of RLS (Nikic et al., 2007). However, in a recent report, iron deficiency, anemia, and calcium were not found to be statistically related to RLS in hemodialysis patients (Wali and Alkhouli, 2015). Previous studies showed conflicts about the association between BMI and RLS in end-stage renal disease patients. A study with a large cohort found that the prevalence of RLS in end-stage renal disease patients was positively related to their BMI (Gao et al., 2009). The OR for RLS was 1.42 (95% CI: 1.3–1.6; *p* < 0.0001) for patients with BMI from 23 to 30 kg/m^2^ and 1.60 (95% CI: 1.5–1.8; *p* < 0.0001) for patients with highest BMI compared with patients with lowest BMI (Gao et al., 2009). Decreased number of dopamine receptors in obese people’s brain was considered to be the possible reason (Wang et al., 2001). However, another previous study revealed no such relationships (Kim et al., 2008). A recent cross-sectional study between control group, renal transplantation group and hemodialysis group found that prevalence in renal transplantation is significantly lower than in hemodialysis patients (Kahvecioglu et al., 2016). RLS is very prevalent in end-stage renal disease patients, and hemodialysis patients with RLS were found to have a higher risk of muscle atrophy (Giannaki et al., 2011), cardio/cerebrovascular events and mortality (Lin C.H. et al., 2015). To prevent more morbidity in end-stage renal disease patients, their RLS should be diagnosed in early stage and receive standard treatment of RLS or have a renal transplantation as soon as possible.

## Pathophysiology

The pathophysiology of RLS is still partially understood. The most accepted pathways include genetics variants, abnormal iron metabolisms, dopaminergic dysfunction, and central opiate system.

### Iron

It’s widely accepted that the local brain iron level plays an important role in RLS pathophysiology, however, the mechanism is still unclear. Recent studies demonstrated that the iron deficiency in brain was related to the function of blood-brain interface, as BBBs endothelial cells acted as an iron reservoir for the brain. A dysfunction of iron regulatory protein in the microvasculature incited dysregulation of iron transport across the BBB, resulting in a decrease of iron storage in endothelial cells (Lee et al., 2001). Biochemical studies on the effect of iron in brain indicated that several proteins containing iron were included in various processes like oxidative phosphorylation, oxygen transportation, myelin production and the synthesis and metabolism of neurotransmitters (Ward et al., 2014). Therefore, iron deficiency can lead to cellular damage by oxidation and modification of cellular compounds such as lipids, carbohydrates, protein, and DNA from hydroxyl radical production (Ward et al., 2014). The interactions between impaired neuronal iron uptake and the functions of the neuromelanin-containing and dopamine-producing cells play important roles in RLS pathophysiology (Michaud et al., 2004). Decreased extracellular dopamine, DAT, D1 and D2 receptors are found in iron deficiency, indicating that iron affect the brain dopaminergic transmission in different ways (Dauvilliers and Winkelmann, 2013).

### Dopamine

A large number of pharmacological studies and clinical findings have provided evidence for the important role of dopaminergic system dysfunction in RLS (Winkelmann et al., 2001; Paulus and Trenkwalder, 2006; Galbiati et al., 2015). An improvement of RLS symptoms was found in patients receiving low-dose dopaminergic medications (Paulus and Trenkwalder, 2006; Galbiati et al., 2015) while a worsening of RLS symptoms in patients receiving dopamine antagonists (Winkelmann et al., 2001). The need for dopaminergic agonists to cross the BBB to be effective in RLS symptoms indicated that dopaminergic system in central nervous system was entailed in RLS pathophysiology rather than in peripheral nervous system (Garcia-Borreguero and Williams, 2014). Tyrosine hydroxylase is a rate-limiting step enzyme for the conversion of levodopa to dopamine. As iron is a cofactor of this enzyme, iron deficiency can alter the dopaminergic system in the brain (Dauvilliers and Winkelmann, 2013). Dopaminergic A11 cells, located in the midbrain and close to hypothalamus, have long axons and project diffusely throughout the spinal cord (Clemens and Hochman, 2004). They are the major source of dopamine in spinal cord. A11 cells arrive in the dorsal horn, then project to the motoneuronal site (Holstege et al., 1996). It’s found that stereotaxic bilateral 6-hydroxydopamine lesions into the A11 nucleus can result in an increased average number of standing episodes and total standing time comparing to the sham rats (Ondo et al., 2000), suggesting an important role of A11 dopaminergic cells in pathophysiological pathways in RLS. A significantly decreased *N*-acetylaspartate, creatinine ratio, and *N*-acetylaspartate concentrations were found in the medial thalamus of RLS patients in a recent study using proton magnetic resonance spectroscopy (Rizzo et al., 2012). Functional MRI and PET studies concluded an important role of medial thalamic nuclei in RLS pathophysiology. The medial thalamic nuclei are part of the limbic system, which is modulated by dopaminergic afferents. Another study found thalamic activity changes in the thalamocotrical circuit (Goulart et al., 2014). Therefore, it was hypothesized that the dopaminergic dysfunction might lead to an impairment of the medial pain system (Garcia-Borreguero and Williams, 2014; Goulart et al., 2014) and then caused the uncomfortable symptoms of RLS. Pharmacological studies also demonstrated that opioids had a protective effect in some RLS patients. An *in vitro* study in rats found that iron deficiency can cause cell death, mainly dopaminergic cells in substantia nigra and opioids could protect them from cell death under the condition of iron deprivation (Sun et al., 2011). The authors concluded from this result that an intact endogenous opioid system and opioid treatment could prevent the dopamine system from dysfunction in iron deficiency patients (Sun et al., 2011). A study of tsDCS in RLS patients showed supportive evidence of spinal cord hyperexcitability (Heide et al., 2014).

### Genes

Studies demonstrate a strong relationship between genetic predisposition and early-onset RLS, of which more than 60% reveal a positive familial history. Moreover, inheritance was found to be related to late-onset RLS and secondary RLS (Winkelmann et al., 2000; Xiong et al., 2010; Yang et al., 2011). A study among 249 Canadian RLS patients found a family history existed in 77.1% of them while the rest 22.9% were sporadic (Yang et al., 2011). Variants in *MEIS1*, *BTBD9* and *MAP2K5*/*SKOR1* resulted in a much higher risk of RLS among a US population (Yang et al., 2011). So far, the GWASs have reported the following susceptible SNPs in association with RLS: *MEIS1* (chromosome 2p14), *BTBD9* (chromosome 6p21.2), *PTPRD* (chromosome 9p24.1-p23), *MAP2K5*/*SCOR1* (chromosome 15q23) and chromosome 16q12.1 (Berger et al., 2002; Hogl et al., 2005; Stefansson et al., 2007; Schormair et al., 2008). Main function of these genes was related to embryonic neuronal development and limb development (Dauvilliers and Winkelmann, 2013; Garcia-Borreguero and Williams, 2014). In RLS autopsy cases, *MEIS1* gene was found to be associated with an increase in H-ferritin, L-ferritin and divalent metal transporter-1 RNA expression in the thalamus (Catoire et al., 2011), suggesting *MEIS1* gene mutants predisposed to lower iron condition. Another study used RNA interference techniques in a lymphoblastoid cell line in which the *MEIS* mRNA expression was blocked (Silver et al., 2010). Forty-eight hours later, an increase in transferring-2 receptor, ferroportin mRNA and *BTBD9*, and a decrease in hepcidin mRNA expression were observed (Silver et al., 2010), indicating that *MEIS1* controlled cellular iron transfer to mitochondria, cellular export of iron and potentially affected *BTBD9* expression and its downstream iron modulation. The effect of *MEIS1* on iron homeostasis was also shown in *Caenorhabditis elegans* (Catoire et al., 2011). A study using *dBTBD9* mutant flies showed significantly decreased brain dopamine and abnormal sleep phenotype which was completely retrieved by administering with pramipexole, a dopamine D2 receptor agonist (Freeman et al., 2012). Furthermore, RLS symptoms can be reconstructed by knocking-down *dBTBD9* expression. While over expression of *BTBD9* in HEK cells showed that the gene controlled iron homeostasis through the regulation of IRP2 (Freeman et al., 2012). The authors suggested that the *BTBD9* protein belonged to a protein family which contained substrate adaptors for the Cul3 class (Freeman et al., 2012). Cul3 class of E3 ubiquitin ligases was shown to regulate sleep in flies (Stavropoulos and Young, 2011). A susceptible view point was that *BTBD9* played a role in dopamine biosynthesis by unclear mechanisms (Shaw and Duntley, 2012). These SNPs had strong association with PLM in RLS patients (Moore et al., 2014). The exact pathophysiological pathways of these genes still remain unclear. An SNP study in the Spanish Caucasian population suggested a modest but significant association between vitamin D receptor *rs731236* SNP and the risk for RLS. RLS patients carrying the allelic variant *rs731236G* had an earlier onset age while those carrying the allelic variant *rs731236GG* had more severe symptoms (Jimenez-Jimenez et al., 2015). A weak association between heme oxygenase genetic variants HMOX1 *rs2071746* polymorphism and the risk of developing RLS was found in the Spanish population (Garcia-Martin et al., 2015). Linkage analyses in families identified several genetic loci for RLS: *RLS1* (chromosome 12q12–q21), *RLS2* (14q13–q21), *RLS3* (9p24–p22), *RLS4* (2q33), *RLS5* (20p13), *RLS6* (19p13), and *RLS7* (16p12.1) (Desautels et al., 2001, 2005; Bonati et al., 2003; Kemlink et al., 2007). The authors suggested the inheritance mode of RLS could be a recessive mode or a dominant mode with variable of inheritance. The main SNP findings are summarized as below (**Table 3**).

**Table 3:** Main SNP findings associated with RLS.

	Loci	Protein	Function
MEIS1	2p14	Homeodomain transcription factor	Iron transportation, BTBD9 regulation (Silver et al., 2010; Catoire et al., 2011)
BTBD9	6p21.2	Zinc finger transcription factor	Dopamine biosynthesis, sleep regulation, regulation of IRP2 (Freeman et al., 2012)
PTPRD	9p24.1-p23	Type IIa receptor-like protein tyrosine phosphatases	Axon guidance and termination of mammalian motorneurons during embryonic development (Uetani et al., 2006)
MAP2K5/LBXCOR1	15q23	Mitogen-activated protein kinase/homeodomain transcription factor	Neuroprotection of dopaminergic neurons (Paulus et al., 2007), development of sensory pathways in the spinal cord that relay pain and touch (Winkelmann et al., 2007)

### Nervous System Structures

Previous theories about dopaminergic effect on RLS pathophysiology mainly focused on substantia nigra and dopaminergic A11 cell group (Ondo et al., 2000; Trenkwalder and Paulus, 2010), nevertheless, changes in dopaminergic neurons in basal ganglia in RLS patients were also found in autopsy studies. This result might be related to iron deficiency in brain (Connor et al., 2003). Dopaminergic projections to the spinal cord originate exclusively from A11 cell group (Noga et al., 2004). Dopamine acted as an excitatory and inhibitory neurotransmitter in spinal cord to regulate sensory, motor as well as autonomic functions (Dauvilliers and Winkelmann, 2013). Recent studies reported frequent PLMS in patients with spinal cord injury, indicating the center role of spinal cord in RLS pathophysiology process (Dauvilliers and Winkelmann, 2013). It might also result from the decreasing supraspinal inhibition to the spinal cord. Functional MRI studies showed changes of thalamic and cerebral activation in RLS (Bucher et al., 1997). However, postmortem studies in RLS patients showed no evidence of changes in the volume of tyrosine kinase (+) neurons or gliosis changes in A11 region in the posterior hypothalamus (Earley et al., 2009). This might result from the fact that only six cases are included in this study or the hypothesis that the manifestations of RLS may be secondary to dopamine metabolism or changes in the distal A11 synapses which is not as easily detected as structural or quantity changes in the cell bodies (Earley et al., 2009). Another recent study indicated that domperidone, a dopamine antagonist that cannot cross the BBB increased the frequency of RLS in patients with Parkinson’s Disease, proposing that peripheral dopaminergic neurons might play an important role in RLS (Rios Romenets et al., 2013). It’s widely believed that iron deficiency in brain causes the decrease in dopaminergic function which then motivates spinal hyperexcitability, leading to the spontaneous sensory and motor movements of RLS (Garcia-Borreguero and Williams, 2014).

## Treatment

Treatment before RLS, three aspects should be considered: lifestyle change, medication effect and iron deficiency (defined as ferritin < 75 ng/mL or iron/TIBC ratio < 20%) (Mackie and Winkelman, 2015). Lifestyle like sleep deprivation, alcohol or tobacco use, decreased motility, or in medication (dopamine antagonists, antihistamines or serotonergic antidepressants, opioid discontinuation or blood loss) can result in earlier onset or increase severity in RLS symptoms. A detailed inquiry for the patient should be carried out. Treatment of RLS mainly include pharmacological and non-pharmacological treatment.

### Pharmacological Treatment

Dopaminergic agents are considered to be the first-line treatment for RLS, including pramipexole and ropinirole (Garcia-Borreguero et al., 2013; Silber et al., 2013). Pergolide and cabergoline are not recommended due to their association with increased risk of valvular heart disease (Zanettini et al., 2007). Ropinirole has a faster onset with shorter duration, while rotigotine is commonly used as a transdermal patch which continuously provides stable plasma drug concentrations, resulting in its particular therapeutic effect on patients with symptoms throughout the day (Mackie and Winkelman, 2015). α2δ agonists have become increasingly important in treating RLS, for being considered as possible first-line agents for RLS. A recent double-blind study over 12-week period compared the efficacy of pregabalin, pramipexole and placebo, demonstrating a better efficacy of pregabalin rather than dopamine agonist or placebo (Hubner et al., 2013). Moreover, the difference of the efficacy varied with drug doses (Hubner et al., 2013). Opioids have been found to be effective in treating RLS, but the potential drug abuse and side effects including respiratory depression and constipation limit its use in RLS, as they are not commonly advised as initial treatment of choice. A recent study on treating first-line agents refractory RLS with extended-release oxycodone–naloxone combination showed very impressive and persistent effect of this combination on RLS symptoms (Trenkwalder et al., 2013). Other pharmacological treatments include iron supplement, some other anticonvulsants and benzodiazepines. The choice of two major first-line agents should be considered with their side effects. Dopamine agonists can cause somnolence and ICDs like compulsive gambling or over-eating, while common side effects of α2δ agonists are weight gain, dizziness and gait instability. As a result, for initial treatment of RLS patients, dopaminergic agents are used as first-line agents in patients with very severe symptoms, over-weighted, comorbid depression, risk of falls, or cognitive impairment (Garcia-Borreguero et al., 2013), while α2δ agonists are advised as first-line agents in patients with severe sleep disturbance, comorbid anxiety, RLS-related pain, or previous history of ICDs (Garcia-Borreguero et al., 2013). Evidence-based guidelines on treating RLS were published by EFNS and IRLSSG in 2012 and 2013 respectively. Many other pharmacological treatment reviews and research papers of RLS have been published recently (Garcia-Borreguero and Williams, 2014; Hornyak et al., 2014). A conclusion of several medications is shown above (**Table 4**).

**Table 4:** Recommendation drugs for RLS.

Drug			Recommendation level		Average dose (mg/day)	Common side effects
			Short-term^a^	Long-term^b^		
Dopaminergic agents	Non-ergot derived	Pramipexole	A	B	0.125–0.75	Sleepiness, nausea, and insomnia
		Ropinirole	A	B	0.25–4.0	Nausea, headache, fatigue, dizziness, and vomiting
		Rotigotine	A	B	0.5, 1, 2, or 3	Application-site reactions to the patch, nausea, headache, and fatigue
	Ergot derived	Pergolide	Only used in RLS refractory to all other treatments		*c*	Very serious side effects: fibrosis and valvulopathy
		Cabergoline			
	Levodopa		A	B	≤200	Nausea, augmentation, and loss of efficacy
α2δagonists	Gabapentin enacarbil		A	A	600–1800	Somnolence, dizziness, and headache
	Pregabalin		A	*d*	≈300	Dizziness, somnolence, fatigue, and nausea

*^a^Short-term treatment 6 months; ^b^Long-term treatment ≥ 1 year; ^c^Not well-known; ^d^nsufficient evidence.*

Other drugs such as dopaminergic agents (piribedil), anticonvulsants (gabapentin), opioids (tramadol, methodone), iron, hypnotic and sedative agents, folate, vitamin B_12_, magnesium, vitamin E, botulinum toxin, physiotherapy, phototherapy, and aerobic exercises are not recommended in clinical practice due to insufficient evidence. However, they can be used as auxiliary drugs concerning to the symptoms and comorbidities of the patient.

As depression is a very common comorbidity in RLS and many antidepressants such as selective serotonin reuptake inhibitors (SSRIs) and tricyclic antidepressants (TCAs) can worsen the symptoms of RLS, treatment of depression in RLS patients should be cautious. Since bupropion, a newer antidepressant, doesn’t show any evidence of exacerbation of RLS symptoms, it is used as an effective antidepressant in these patients (Mackie and Winkelman, 2015).

Restless legs syndrome is very common in women during pregnancy and lactation, with a prevalence of 15–25% (Hubner et al., 2013). Guidelines on treating RLS during pregnancy and lactation have been published by IRLSSG in 2014 (Picchietti et al., 2015) (**Table 5**). Once RLS is diagnosed in pregnancy, non-medicine treatment should be considered firstly. Patients should be educated about the natural course of RLS during pregnancy, in which RLS commonly remits or disappears after delivery. Moderate exercise and avoidance of aggravated factors such as iron deficiency, long-term immobility, serotonergic antidepressants should be suggested. Iron level should be measured to decide whether to treat the patient with iron. Iron therapy (oral or intravenous) should be given when serum ferritin < 75 ug/L. When serum ferritin > 75 ug/L with refractory RLS, drug treatment should be considered.

**Table 5:** Medication treatment for RLS in women during pregnancy and lactation.

Women during pregnancy	Cabidopa 25–50 mg or levodopa 100–200 mg in the evening or at night.
	Clonazepam 0.25–1 mg in the evening.
	In very severe or refractory cases: low-dose oxycodone.
	Reassess need for medication periodically and at delivery.
Women during	Reassess iron status.
lactation	Gabapentin 300–900 mg in the evening or at night.
	Clonazepam 0.25–1 mg in the evening.
	In very severe or refractory cases: low-dose tramadol.

Loss of efficacy and augmentation are two main treatment failures after a long course of treatment of RLS. The probable reasons of them might be the natural worsening of RLS symptoms over time or compensatory response of CNS to chronic drug treatment (Mackie and Winkelman, 2015). Iron deficiency should be treated with iron supplement. Both of the intravenous and oral iron formulations are proved to be effective in some RLS patients (Trotti et al., 2012; Hornyak et al., 2014). Further study about effect of iron therapy in all RLS patients or only a certain type of RLS remains to be investigated.

Augmentation refers to a worsening of the symptoms that occurs very commonly among RLS patients after long-term treatment with some certain medications. An overall augmentation rate of 5.6% was reported (Liu et al., 2016). All the current dopaminergic drugs and another non-dopaminergic drug tramadol have been reported to show some degree of augmentation (Trenkwalder et al., 2007; Vetrugno et al., 2007; Hogl et al., 2010; Oertel et al., 2011), which are thought to be related to dose and duration of medication and individual factors such as iron deficiency (Garcia-Borreguero et al., 2013). The highest incidence rate of augmentation occurred with levodopa was up to 60–80% in RLS patients (Hogl et al., 2010). In addition, incidence rate was reported to be higher in patients treating with shorter-acting dopaminergic agents (pramipexole, ropinirole) than longer-acting dopaminergic agents (rotigotine, cabergoling). A possible explanation is the masking of earlier symptom onset by longer-acting dopaminergic agents (Garcia-Borreguero and Williams, 2014). To prevent augmentation, it is important to initiate the treatment with α2δ agonists for milder RLS patients (Silber et al., 2013), and a lowest effective dose of dopaminergic agents should be established to decrease the incidence and delay the occurrence of augmentation (Silber et al., 2013). Management of augmentation is not to increase the dose of dopaminergic agents, but to add a non-dopaminergic agent as a combination strategy (Silber et al., 2013). Moreover, loss of efficacy is the reduction of drug efficacy over time. In these cases, RLS symptoms are not worse than before initiating the treatment (Garcia-Borreguero and Williams, 2014), a combination therapy is recommended in loss of efficacy to decrease the side effects of certain medications, as well as to prevent augmentation.

### Non-pharmacological Treatment

Sleep hygiene should be corrected before all the pharmacological treatment. Sleep deprivation, sleep disturbances and factors that can result in insomnia should all be avoided. Another common but easily neglected disorder is OSAS. Early treatment for OSAS is beneficial for improving sleep for RLS patients. Other non-pharmacological treatments have been proven to be effective in RLS. tsDCS showed a short-lasting clinical improvement in idiopathic RLS patients (Heide et al., 2014), while high-frequency rTMS resulted in an significant improvement in the motor symptoms and sleep disturbances in RLS patients (Lin Y.C. et al., 2015). But these non-pharmacological treatment for RLS studies are scarce. They show some advantages in symptomatic RLS patients who do not respond to or do not tolerate the classic pharmacological treatments. It may bring brand new solutions to these patients. On the other hand, these methods are non-invasive and safe, no significant side effects have been observed yet. Developing these new methods can be a great benefit for RLS patients.

## Conclusion

There have been a lot of advances in the fields of RLS in recent years, as the diagnostic criteria have been revised in 2012. A deeper insight in pathophysiology of RLS has put iron metabolism dysfunction in an important position, as well as dopaminergic system dysfunction, has been demonstrated. Guidelines of long-term treatment of RLS are published by IRLSSG in 2013 to help with the treatment of RLS for clinicians. Prevention and management of augmentation for long-term treatment has been improved through clinical experience and researches. More researches should be done to discover the pathophysiology, and to find better treatment and management of augmentation of RLS.

## Author Contributions

SG: drafting the manuscript and the tables; HJ, CH, JL, XX, GZ: giving advice on conception and design; JH, NX: revising the manuscript; ZL: giving advice on conception and design; TW: conception and design. Giving final approval of the version to be published.

## Conflict of Interest Statement

The authors declare that the research was conducted in the absence of any commercial or financial relationships that could be construed as a potential conflict of interest.

## Abbreviations

ADHD: attention-deficit/hyperactivity disorder
AS: ankylosing spondylitis
BBBs: blood–brain barriers
CH-RLSq: Cambridge–Hopkins diagnostic questionnaire for RLS
CI: confidence interval
CSF: cerebrospinal fluid
Cul3: Cullin-3
DA: dopaminergic
DAT: dopamine transporter
DCSAD: Diagnostic Classification of Sleep and Arousal Disorders
DIMS: disorder of initiating and maintaining sleep
DOES: disorder of excessive somnolence
EFNS: European Federation of Neurological Societies
EXP: expanded screening questionnaire
GWASs: genome-wide association studies
HRQoL: health-related quality of life
ICDs: impulse control disorders
ICSD: International Classification of Sleep Disorders
IRLS: international restless legs scale
IRLSSG: International Restless Legs Syndrome Study Group
IRP2: iron regulatory protein-2
NIH: National Institutes of Health
OR: odd ratio
OSAS: obstructive sleep apnea syndrome
PLMs: periodic limb movements
PLMS: periodic limb movement during sleep
PLMW: periodic limb movement during wakefulness
PSG: polysomnogram
RLS: restless legs syndrome
rTMS: repetitive transcranial magnetic stimulation
SNPs: single nucleotide polymorphisms
tsDCS: transcutaneous spinal direct current stimulation.
